# Transcriptomic responses of *Microcystis aeruginosa* under electromagnetic radiation exposure

**DOI:** 10.1038/s41598-020-80830-z

**Published:** 2021-01-22

**Authors:** Chao Tang, Ziyan Zhang, Shen Tian, Peng Cai

**Affiliations:** 1 Physical Environment Group, Key Laboratory of Urban Environment and Health, Institute of Urban Environment, Chinese Academy of Sciences, 1799 Jimei Road, Xiamen, 361021 People’s Republic of China; 2 Xiamen Key Laboratory of Physical Environment, 1799 Jimei Road, Xiamen, 361021 People’s Republic of China; 3 Shanghai Institute of Nutrition and Health, Chinese Academy of Sciences, 320 Yueyang Road, Shanghai, 200031 People’s Republic of China

**Keywords:** Transcriptomics, Environmental impact, Mechanism of action

## Abstract

Electromagnetic radiation is an important environmental factor. It has a potential threat to public health and ecological environment. However, the mechanism by which electromagnetic radiation exerts these biological effects remains unclear. In this study, the effect of *Microcystis aeruginosa* under electromagnetic radiation (1.8 GHz, 40 V/m) was studied by using transcriptomics. A total of 306 differentially expressed genes, including 121 upregulated and 185 downregulated genes, were obtained in this study. The differentially expressed genes were significantly enriched in the ribosome, oxidative phosphorylation and carbon fixation pathways, indicating that electromagnetic radiation may inhibit protein synthesis and affect cyanobacterial energy metabolism and photosynthesis. The total ATP synthase activity and ATP content significantly increased, whereas H^+^K^+^-ATPase activity showed no significant changes. Our results suggest that the energy metabolism pathway may respond positively to electromagnetic radiation. In the future, systematic studies on the effects of electromagnetic radiation based on different intensities, frequencies, and exposure times are warranted; to deeply understand and reveal the target and mechanism of action of electromagnetic exposure on organisms.

## Introduction

Water bloom not only causes serious disasters to the ecosystem, but also poses a huge safety hazard to people's drinking water. *Microcystis aeruginosa* (*M. aeruginosa*) is one of the most common cyanobacterial blooms. *M. aeruginosa* produces microcystins, which have typical biotoxic effects and can cause serious biosafety hazards^1^. The effects of chemical substances, such as heavy metal and antibiotics, on the growth of cyanobacteria have been widely reported^2,3^. Generally, low concentrations of heavy metals and antibiotics promote algal growth, whereas high concentrations have the opposite effects^4–7^.

With the rapid development of the electromagnetic environment, excessive electromagnetic radiation has been found to exert potential hazards to animals and plants^8–11^. Some studies have reported on the effects of the electromagnetic environment on algal growth. One Study has shown that a specific electromagnetic wave can increase *Spirulina platensis* chlorophyll content, photosynthetic rate, and biomass^12^. In our previous study, we found that oxidative stress of *M. aeruginosa* can be induced under electromagnetic radiation, and regulations on key enzymes of photosynthesis (Rubisco and fructose-1,6-bisphosphate aldolase (FBA)) by electromagnetic radiation indicated that electromagnetic radiation can affect the photosynthesis of *M. aeruginosa*^13^.

Some studies have eliminated algae using electromagnetic fields. The pulsed electric field has a significant inhibitory effect on algal growth, and the electric field intensity is the most important impact factor as increasing it can significantly enhance the inhibitory effect on algal growth^14^. The experimental results indicated that the pulsed magnetic field can kill algae; increase intensity, retention time and that the pulse frequency can enhance the efficiency^15^. Electromagnetic waves can cause molecular vibrations in algal cells, causing their cell walls to rupture and leading to accumulation of cellular contents, which ultimately leads to cell death^16^. The influence of electromagnetic radiation on algal growth indicates that the electromagnetic environment may be correlated with aquatic environment safety, which is worthy of further exploration.

Proteomic and transcriptomic methods have been successfully applied to cyanobacteria. These methods have been used to understand the mechanisms of environmental stress (such as antibiotics^17^, metal ions^18^, organic pollutants^19^, nitrogen limitation^20^) on algae at the molecular level. However, the effects of electromagnetic radiation on the growth of cyanobacteria at the molecular level remain to be elucidated. In our previous study using proteomics, electromagnetic radiation altered the expression levels of photosynthesis-related proteins, and we speculated that the photoreaction system may be the target of electromagnetic radiation on cyanobacteria^21^. To further determine the effect of electromagnetic radiation on the growth of cyanobacteria, the present study investigated the effects of 1.8 GHz (the most common and widely used mobile communication frequency in mainland China is 1.8 GHz) and 40 V/m electromagnetic radiation on cyanobacteria cells through transcriptomic methods and measured the activity of ATP synthase. Analyze the influence mechanism of the electromagnetic environment on algae growth and explore the methods and means of physical to control algae growth rate, will be of great scientific significance and practical significance to evaluate the potential ecological risk of electromagnetic environment and solve the increasingly serious problem of water bloom.

## Results

### Differentially expressed genes analysis

**Figure 1:**
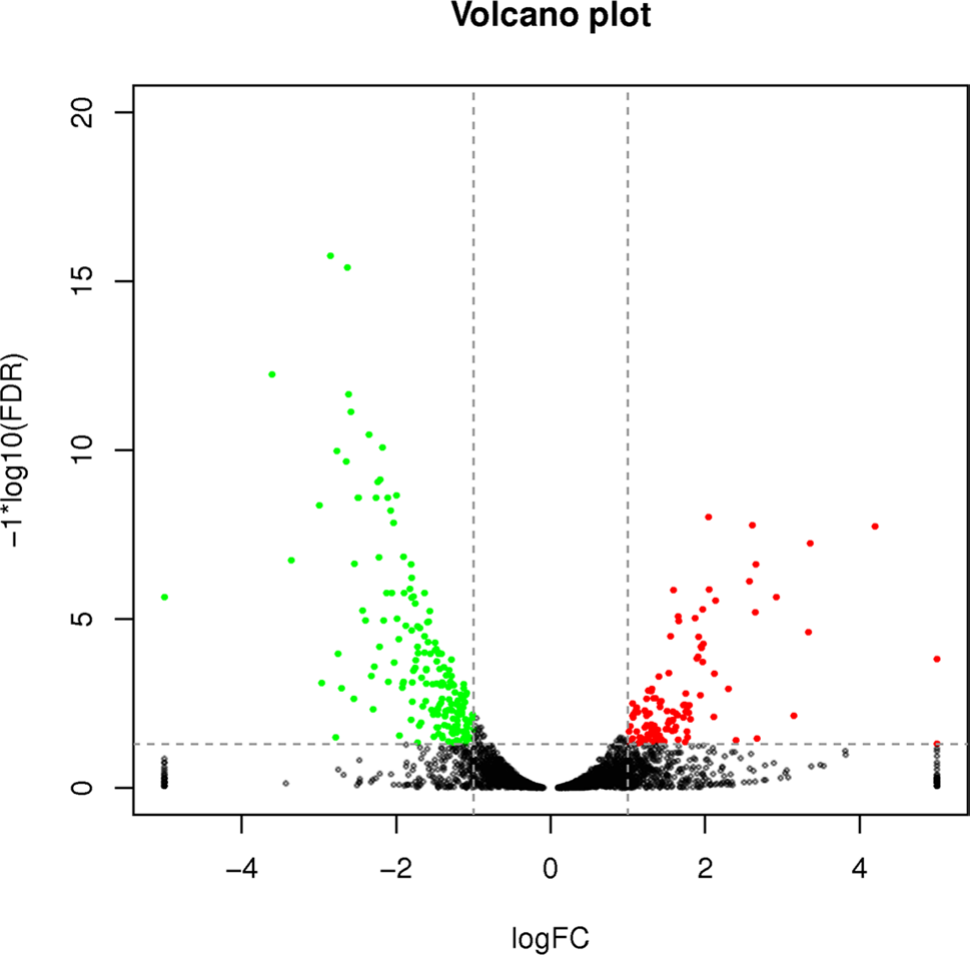
Volcanic plot of C versus E (The control group mark was C and the electromagnetic radiation exposure group mark was E. The abscissa indicates the logarithm value of the difference multiple between E and C. The ordinate indicates the negative Log10 value of the FDR of the difference between the two groups. The red (E relative to C, gene expression is upregulated) and green (E relative to C, gene expression is downregulated) dots indicate the difference in gene expression (the criterion is FDR < 0.05, and the difference multiple is more than twice), and the black points are not different).

Between the treatment and control groups, 306 differentially expressed genes were determined, 121 and 185 of which were upregulated and downregulated, respectively (Fig. 1). The full list of differentially expressed genes with the fold change and false discovery rate (FDR) detailed can be found as Supplementary Table S1.

### Pathway enrichment analysis of differentially expressed genes

Exposure of *M. aeruginosa* to electromagnetic radiation caused significant differential gene expression and corresponding enrichment of the ribosome, oxidative phosphorylation and carbon fixation in photosynthetic pathways of organisms (Table 1).

**Table 1:** Differentially expressed genes enrichment KEGG Pathway.

Number	Pathway	DEGs genes with pathway annotation (115)	All genes with pathway annotation (989)	*P* value (%)	*Q* value	Pathway ID
1	Ribosome	16 (13.91%)	54 (5.46%)	0.02	0.01	ko03010
2	Oxidative phosphorylation	14 (12.17%)	47 (4.75%)	0.05	0.02	ko00190
3	Carbon fixation in photosynthetic organisms	7 (6.09%)	17 (1.72%)	0.17	0.04	ko00710
4	Glycolysis/gluconeogenesis	10 (8.7%)	35 (3.54%)	0.45	0.08	ko00010
5	Pentose phosphate pathway	6 (5.22%)	17 (1.72%)	0.91	0.13	ko00030
6	RNA polymerase	3 (2.61%)	5 (0.51%)	1.29	0.13	ko03020
7	Photosynthesis	14 (12.17%)	66 (6.67%)	1.48	0.13	ko00195
8	Starch and sucrose metabolism	7 (6.09%)	24 (2.43%)	1.53	0.13	ko00500
9	Carbon metabolism	15 (13.04%)	78 (7.89%)	2.82	0.22	ko01200
10	Photosynthesis—antenna proteins	4 (3.48%)	12 (1.21%)	4.10	0.29	ko00196
11	Nitrogen metabolism	5 (4.35%)	18 (1.82%)	4.82	0.31	ko00910

The second column is the pathways; the third column is the number of differentially expressed genes that are noted to a pathway and the percentage of differentially expressed genes to the total number of differentially expressed genes (number of headings); the fourth is the number of genes annotated to a pathway and the percentage of genes to the total number of genes (number of headings); the fifth column is the *P* value; the sixth column is the Q value after multiple checks; the seventh column is the pathway ID.

Sixteen differentially expressed genes were present on the ribosome pathway under electromagnetic stress. The corresponding differentially expressed genes are listed in Table 2. The expression of genes regulating 30S ribosomal proteins S3, S8, S9, S10, S17, and S19 and 50S ribosomal proteins L2, L5, L6, L13, L14, L15, L16, L22, L24, and L29 was downregulated.

**Table 2:** Differentially expressed genes enrichment Pathway.

Gene	Protein		log_2_(FC)	
**Differentially expressed genes enrichment ribosome Pathway**				
rpsJ	30S ribosomal protein S10		− 2.54	
rplB	50S ribosomal protein L2		− 1.21	
rpsS	30S ribosomal protein S19		− 1.33	
rplV	50S ribosomal protein L22		− 1.80	
rpsC	30S ribosomal protein S3		− 2.00	
rplP	50S ribosomal protein L16		− 1.75	
rpmC	50S ribosomal protein L29		− 2.43	
rpsQ	30S ribosomal protein S17		− 2.11	
rplN	50S ribosomal protein L14		− 2.35	
rplX	50S ribosomal protein L24		− 2.16	
rplE	50S ribosomal protein L5		− 2.21	
rpsH	30S ribosomal protein S8		− 1.66	
rplF	50S ribosomal protein L6		− 1.61	
rplO	50S ribosomal protein L15		− 1.18	
rplM	50S ribosomal protein L13		− 2.65	
rpsI	30S ribosomal protein S9		− 2.22	
**Differentially expressed genes enrichment oxidative phosphorylation pathway**				
ndhF3	NAD(P)H dehydrogenase, subunit NdhF3 family protein		− 1.91	
ndhD4	Proton-translocating NADH-quinone oxidoreductase, chain M family protein		− 2.77	
atpG	ATP synthase subunit b'		− 2.22	
atpF	ATP synthase subunit b		− 2.58	
atpH	ATP synthase subunit delta		− 2.50	
atpA	ATP synthase subunit alpha		− 2.18	
atpG	ATP synthase gamma chain		− 1.72	
ndhB	NAD(P)H-quinone oxidoreductase subunit 2		− 1.13	
atpC	ATP synthase epsilon chain		− 1.75	
atpD	ATP synthase subunit beta		− 1.28	
sdhA	Succinate dehydrogenase/fumarate reductase, flavoprotein subunit		1.24	
ndhA	NAD(P)H-quinone oxidoreductase subunit 1		− 1.26	
ndhI	NAD(P)H-quinone oxidoreductase subunit I		− 1.78	
ndhG	NADH-ubiquinone/plastoquinone oxidoreductase chain 6 family protein		− 2.49	
**Differentially expressed genes enrichment carbon fixation pathway**				
gap2	Glyceraldehyde-3-phosphate dehydrogenase		− 1.22	
Fba	Fructose-bisphosphate aldolase, class II, Calvin cycle subtype		− 1.69	
glpX	D-fructose 1,6-bisphosphatase class 2/sedoheptulose 1,7-bisphosphatase		− 1.45	
Tkt	Transketolase		− 2.04	
PRK	Phosphoribulokinase		− 1.36	
Rpe	Ribulose-phosphate 3-epimerase		− 1.54	
pckA	Phosphoenolpyruvate carboxykinase (ATP)		1.43	

Log_2_ (FC) represents the logarithm of genes with a fold change between E and C, with 2 as the base. If FDR < 0.05 and | log_2_FC |> 1, the difference is significant. All genes in this table FDR < 0.05.

Fourteen differentially expressed genes were present on the oxidative phosphorylation pathway of *M. aeruginosa* under electromagnetic stress. Among them, 13 were downregulated, and 1 was upregulated. The corresponding differentially expressed genes are listed in Table 2. The expression of genes regulating NAD(P)H dehydrogenase, subunit NdhF3 family protein, proton-translocating NADH-quinone oxidoreductase, chain M family protein, NADH-ubiquinone/plastoquinone oxidoreductase chain 6 family protein, and NAD(P)H-quinone oxidoreductase subunit 1, 2, I was downregulated. Moreover, the expression of genes regulating ATP synthase subunit alpha, b', b, beta, delta, gamma, and epsilon chains was downregulated. By contrast, the expression of genes regulating succinate dehydrogenase/fumarate reductase and flavoprotein subunit was upregulated.

Seven differentially expressed genes were related to carbon fixation. Among them, 6 were downregulated, and 1 was upregulated. As shown in Table 2, the expression of genes regulating glyceraldehyde-3-phosphate dehydrogenase, fructose-bisphosphate aldolase class 2, Calvin cycle subtype, d-fructose 1,6-bisphosphatase class 2/sedoheptulose 1,7-bisphosphatase, transketolase, phosphoribulokinase, and ribulose-phosphate 3-epimerase was downregulated. By contrast, the expression of genes regulating phosphoenolpyruvate carboxykinase was upregulated.

Moreover, the differentially expressed genes were mainly related to glycolysis/gluconeogenesis, pentose phosphate pathway, RNA polymerase, photosynthesis, starch and sucrose metabolism, carbon metabolism, photosynthesis—antenna proteins, nitrogen metabolism (Table 1).

### Genes and proteins with significant differences

Combined with the results of our previous proteomic study^21^, five genes or proteins showed simultaneous changes in gene and protein levels after electromagnetic radiation. The expression of the genes C789_RS04790, C789_RS04815 was downregulated, whereas that of iron stress-induced chlorophyll-binding protein was upregulated. The gene and protein levels of ATP synthase subunit b showed a downward trend. The expression of two unknown functional genes and proteins was upregulated. The corresponding differentially expressed genes and proteins are listed in Table 3.

**Table 3:** Differentially expressed genes and proteins.

Gene	Protein	Gene log_2_(FC)	Protein log_2_(FC)
C789_RS04790	Iron stress-induced chlorophyll-binding protein	− 1.37	0.56
C789_RS04815	Iron stress-induced chlorophyll-binding protein	− 1.37	0.56
C789_RS08505	ATP synthase subunit b	− 2.22	− 0.51
C789_RS20075	Uncharacterized protein	1.53	0.30
C789_RS03495	Uncharacterized protein	1.07	0.31

Log_2_ (FC) represents the logarithm of genes or protein with a fold change between E and C, with 2 as the base. Gene: If FDR < 0.05 and | log_2_FC |> 1, the difference is significant. Proteins with fold change in a comparison > 1.2, the difference is significant. All genes or proteins in this table FDR < 0.05.

### Effect of electromagnetic radiation on ATP synthase

**Figure 2:**
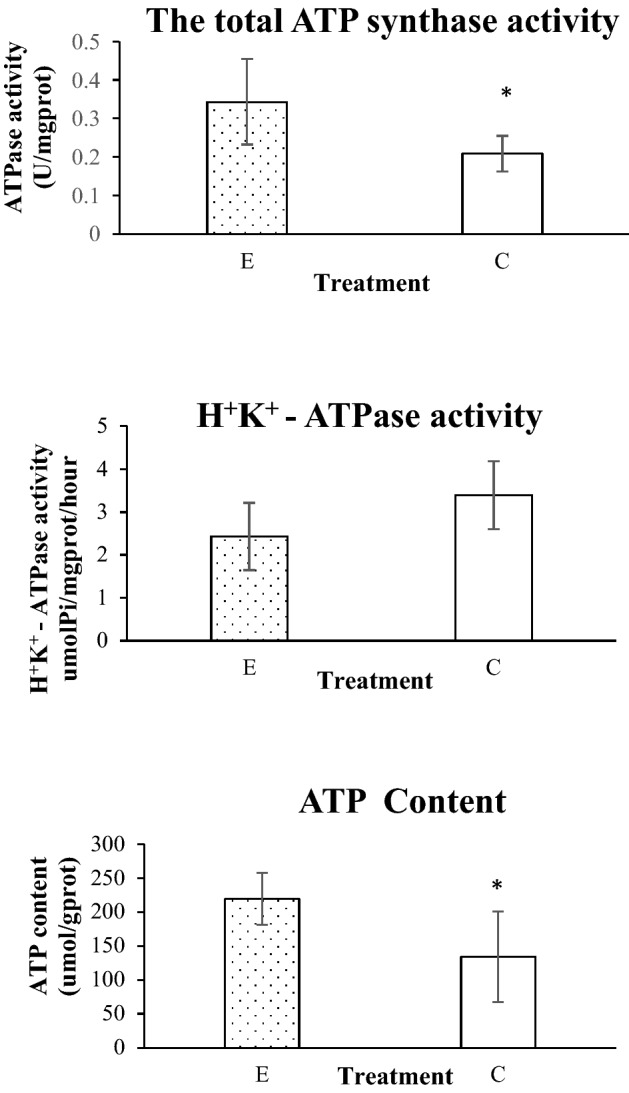
Changes of the total ATP synthase activity, H^+^K^+^-ATP synthase activity and ATP content (The control group mark was C and the electromagnetic radiation exposure group mark was E. Bars represent the SD of the mean, “*” represents *p* < 0.05, E relative to C).

As shown in Fig. 2, 1.8 GHz electromagnetic radiation significantly increased total ATP synthase activity (*p* < 0.05) and ATP content (*p* < 0.05). Electromagnetic radiation reduced H^+^K^+^-ATP synthase activity, but no significance was observed.

## Discussion

Ribosomes are the sites for proteins synthesis. We found that the expression of 16 ribosome-related genes in *M. aeruginosa* was downregulated after electromagnetic radiation, and the number of 50S ribosomal protein-regulated gene was 10, comprising 62.5% of the total ribosome-related differentially expressed genes. 50S ribosomal proteins were more sensitive to electromagnetic radiation than 30S ribosomal proteins. Significantly downregulated genes S10, L2, S19, L22, S3, L16, and L29 belonged to elongation factor thermo unstable (EF-Tu). EF-Tu is a guanosine nucleotide binding protein and plays a central role in protein synthesis, it is responsible for the extension of the peptide chain during protein synthesis^22,23^. EF-Tu forms a quaternary complex with the ribosome with aminoacyl-tRNA and guanosine triphosphate (GTP) and is coupled to GTP hydrolysis. As the aminoacyl-tRNA binds to the ribosome, EF-Tu forms a complex with guanosine diphosphate leaving the ribosome. This process continues to circulate so that the peptide chain continues to extend^24^. EF-Tu also functions as a molecular chaperone; to promote the renaturation of denatured rhodanese, the translation EF-Tu has chaperone-like capacity^25^ When an organism is exposed to environmental stress, the protein denatures. The protein exposure residues aggregate due to interaction, causing irreversible inactivation of the protein. EF-Tu can prevent the aggregation of exposed hydrophilic groups and protect the structure, which returns to normal after the stress conditions disappear, avoiding permanent denaturation^26^. After electromagnetic exposure, the downregulation of genes related to EF-Tu may decrease the amount of EF-Tu synthesis, indicating that electromagnetic radiation may inhibit the peptide chain extension of *M. aeruginosa* cells, affecting protein synthesis, and weaken the function of EF-Tu in preventing protein aggregation.

Cyanobacteria NAD(P)H dehydrogenase is a multi-subunit photosynthetic membrane protein complex located on the thylakoid membrane and plays a vital role in energy metabolism. NAD(P)H-quinone oxidoreductase is involved in many important energy reactions. It was responsible for many functions including respiration, cyclic electron flow around photosystem I (PSI) and CO_2_ uptake^27–29^. Bernát et al. showed that active electron flow from metabolites to plastoquinone is suppressed upon deletion of ndhF1 and PSI-mediated cyclic electron transport is dependent on NdhF3/F4-type NDH-1 complexes^30^. Ogawa showed that the ndhB gene is required for C_i_ transport, the inactivation of the ndhB gene also depressed dark respiration, and NADH dehydrogenase is essential to photoheterotrophic growth and inorganic carbon transport^31,32^. Stress conditions may alter the NAD(P)H dehydrogenase; for example, the contents of NAD(P)H-dehydrogenase are increased in cells grown in an environment with high salinity^33^, high light conditions also regulate NADH genes^34^. Our study shows that electromagnetic radiation also causes downregulation of NAD(P)H-quinone oxidoreductase regulatory gene expression, indicating that electromagnetic radiation may have a certain effect on the NAD(P)H complex of cyanobacteria, which in turn affects a series of physiological processes.

ATP synthase is widely present in the mitochondria and chloroplasts of eukaryotic cells and cytoplasmic membrane of heterotrophic bacteria and photosynthetic bacteria. It participates in oxidative phosphorylation and photosynthetic phosphorylation and catalyzes ATP synthesis under the promotion of transmembrane proton potential. ATP synthase consists of F_o_ unit and F_1_ unit, also known as F_o_F_1_-ATPase, the F_o_ unit acts as a proton channel, whereas F_1_ unit catalyzes ATP synthesis^24^. The following factors affect the function of ATP synthase: ① ATP synthase function is affected through the subunit of F_o_; such as oligomycin binding to the ATP synthase F_o_ subunit, thereby inhibiting H^+^ through F_o_; dicyclohexylcarbodiimide also inhibits the action of protons through F_o_^24^. ② ATP synthase function is affected by the subunit of F_1_; when 1 mol of IF_1_ (the natural ATPase inhibitor from beef heart mitochondria) is bound to 1 mol of F_1_, the ATPase activity is fully inhibited^35^. ③ ATP synthase function is affected by the linkage; Subunit ε is a natural endogenous inhibitor of ATP synthase. It plays a dual role in F_o_F_1_ from bacteria and chloroplasts. Subunit ε is indispensable for coupling between proton translocation though F_o_ and ATP synthesis/hydrolysis in F_1_, and it has a regulatory role inhibiting the ATPase activity of the enzyme^36^. Stimulation of the external environment has a certain effect on ATPase activity, and ATPase is also part of a stress response mechanism of the organism against external stimuli. Plasma membrane H^+^ ATPase mitigates of physiological disturbances imposed by salt press^37^. Our study showed that under the exposure of electromagnetic radiation, the expression of ATP synthase subunit gene was downregulated, it is indicated that electromagnetic radiation may have effects on ATP synthase function. The total ATP synthase activity and the ATP content of cyanobacteria increased significantly. H^+^K^+^-ATPase activity was decreased, but no significant change was observed. This may be because the total ATP synthase contains P-type ATPase, F-type ATPase, and many factors affecting ATPase activity. The specific reasons need further study.

The Calvin cycle of photosynthesis consists mainly of three phases: fixation of CO_2_, reduction of 3-phosphoglycerate, and regeneration of ribulose-1,5-diphosphate. The external environment affects the photosynthetic carbon-fixing enzyme. Studies have shown that suboptimal temperature and low light intensity significantly decreased the growth, photosynthetic rate, activities and mRNA expressions of ribulose 1,5-biphosphate carboxylase/oxygenase, fructose-1,6-bisphosphatase (FBP), glyceraldehyde-3-phosphate dehydrogenase (GAPDH), FBA, transketolase^38^. Cucumber CsGAPDH gene was significantly induced at 2 h after waterlogging treatment, and the peak level of gene expression was observed at 12 h^39^. Adversity environment (high temperature stress, salt stress) will affect the expression of CpFBA gene, creating a “short-term inhibition” effect, where short-term stress will be expressed in large quantities and long-term stress will reduce gene expression levels^40–42^. Studies have also shown that CpFBA exhibits periodic variation under adverse conditions^43^. In our previous study, electromagnetic radiation significantly reduced the activity of FBA enzymes of *M. aeruginosa*^13^. The different response modes of cpFBPase mRNA levels to desiccation stress and high temperature indicated that cpFBPase played an important role in responsing to abiotic stress^44^. The expression of transketolase in rice leaves was upregulated in response to heat stress^45^, whereas the expression of transketolase in *Manihot esculenta Crantz* at 5 and 15 days of drought stress was downregulated^46^. SBPase overexpression enhances photosynthesis under high temperature or salt stress in transgenic rice plants, indicating SBPase plays an important role in plants in stress^47,48^. Studies have shown that the superior water-deficit tolerance in bermudagrass (Tifway) could be mainly associated with Phosphoribulokinase^49^. Phosphoribulokinase was downregulated by drought and abscisic acid in maize (*Zea mays L.*)^50^. A study has shown that phosphoenolpyruvate carboxylase activity was three times higher in infected plant leaves compared to healthy plants^51^. In summary, external stress can affect the gene or protein expression of photosynthetic carbon fixation enzymes or enzyme activity. Our study showed that under the exposure of electromagnetic radiation, seven differentially expressed genes were related to carbon fixation; among them, 6 (gap2, Fba, glpX, Tkt, PRK, Rpe) were downregulated, and 1 (pckA) was upregulated, it indicated that electromagnetic radiation may affect the related synthesis of photosynthetic carbon fixation enzymes through the regulation of gene expression.

After electromagnetic radiation, five genes or proteins showed simultaneous changes in gene level and protein levels. Iron stress-induced protein A (*IsiA*) is the main chlorophyll-binding protein in the thylakoid membrane, and significantly induced under iron deficiency conditions; dynamic changes in *IsiA*-containing complexes during long-term iron deficiency in cyanobacteria may represent an adaptation to iron limitation stress for flexible light energy distribution, which can balance electron transfer between PS I and PS II, thus minimizing photooxidative damage^52^. Havaux also showed that *IsiA* protects cyanobacteria from photooxidativestress^53^. Our results indicate that electromagnetic radiation may affects the function of iron stress-induced chlorophyll-binding protein. In the study, *IsiA* gene expression is downregulated, whereas the corresponding protein levels are increased. We speculate that it may be a negative feedback regulation mechanism. CP43′ protein functions as a nonradiative dissipator of light energy, which is encoded by the *IsiA* gene^54^. Electromagnetic radiation is a kind of energy, which may promote the upregulation of CP43′ protein. The control and exposure groups were placed in the dark for 24 h. The lack of light may cause CP43′ to be downregulated. Electromagnetic radiation upregulated CP43′ protein. In order to keep CP43′ downregulated, the *IsiA* gene is downregulated, forming a negative feedback regulation mechanism. The influence of electromagnetic radiation on *IsiA* gene and related regulatory protein needs further study.

The gene and protein levels of ATP synthase subunit b showed a downward trend after eletcromagnetic exposure. ATP synthase acts on photosynthetic and oxidative phosphorylation to synthesize ATP. ATP synthase b subunit plays an important role in the growth, reproduction and adaptability of organisms. Chen^55^ and Zheng^56^ found that the ATP synthase b subunit gene is related to the testis development of *drosophila*, which will reduce the fecundity of male flies and cause male sterility. Li et al.^57^ found that F_o_ATP synthase b-chain is involved in *white spot syndrome virus* infection. Feng et al. found that the ATP synthase b subunit gene is essential to the survival of rice brown planthopper (BPH), and the RNAi of ATP synthase b shows an effective inhibition effect of the BPH. ATP synthase b may serve as a potential target gene for BPH control^58^. ATP synthase b subunit is essential for normal function of ATP synthase. The gene and protein level of ATP synthase subunit b are downregulated by electromagnetic radiation. This finding indicated that electromagnetic radiation will affect the photosynthesis and energy metabolism of *M. aeruginosa*, which may reduce the ability of these biological processes.

**Figure 3:**
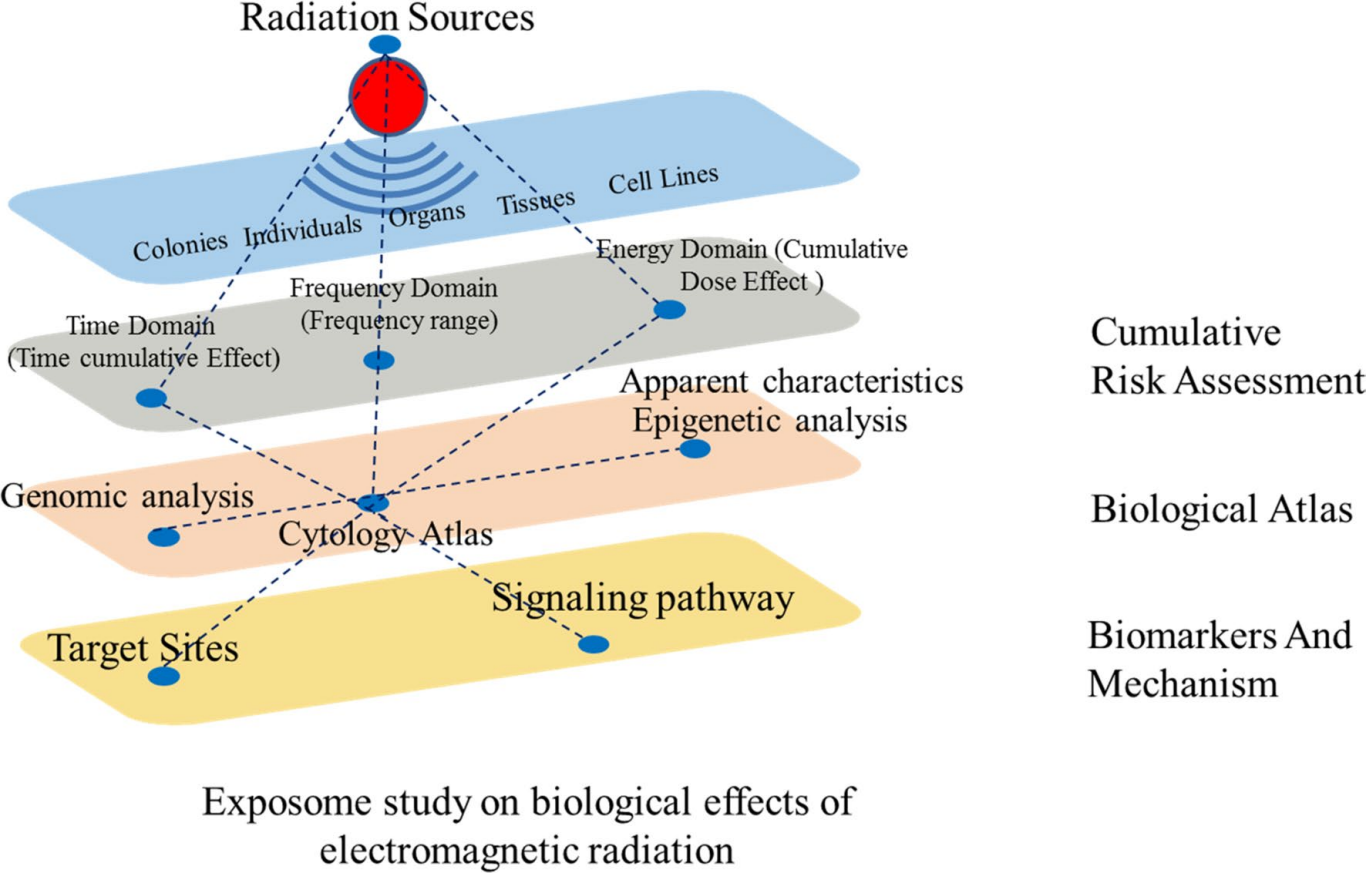
Future research outlook.

This experiment is an exploratory experiment that found some response of cyanobacteria to electromagnetic radiation. Previous studies in our laboratory have found that electromagnetic waves have effects on the energy metabolism of *Caenorhabditis elegans*^59,60^ and yeast^61^, which is similar to the results of this experiment. Next, we need to further study the mechanism of electromagnetic radiation's influence on energy metabolism pathway and changes in energy metabolism-related metabolites and so on. Further experiments investigating the altered pathways would be beneficial to reveal a mechanism by which electromagnetic radiation exerts these biological effects. In the future, it is necessary to base on different intensities (energy domain), frequencies (frequency domain), exposure times (time domain), from the level of cells lines, tissues, organs, individuals, colonies, and adopt different methods (appearance characteristics analysis, epigenetic analysis, genomic analysis, cytology atlas, etc.), systematically study the effects of electromagnetic radiation. It will help the scientific community to deeply understand and reveal the target sites, signaling pathway and mechanism of action of electromagnetic exposure on organisms. Future research outlook is shown in Fig. 3. From study the effects of electromagnetic radiation on organisms to electromagnetic dose effects, clarify the mechanism of electromagnetic radiation, and finally establish electromagnetic radiation protection programs, in order to ensure the safe development of the electromagnetic environment.

In conclusion, electromagnetic radiation affects the expression of ribosome-related regulatory genes, which may inhibit the protein synthesis of *M. aeruginosa*. Electromagnetic radiation affects the expression of Calvin cycle-related regulatory genes, and alters photoreaction system related protein expression levels, thereby disturbing the photosynthesis of *M. aeruginosa*. Moreover, it influences oxidative phosphorylation, thereby affecting cyanobacterial energy metabolism. Our finding suggests that energy metabolism pathway may respond positively to electromagnetic radiation.

## Materials and methods

### Experimental materials

The experiment involved species from *M. aeruginosa* (FACHB-905), and came from the Institute of Aquatic Biology, Chinese Academy of Sciences. The culture medium was BG 11 medium^21^.

### Experimental treatment

*M. aeruginosa* were cultured in the normal light (Culture temperature was 25 ± 1 °C with 12 L: 12 D light–to-dark ratio, light intensity was 1000 ± 100 lx) for a period. Then, *M. aeruginosa* suspension were divided into two parts for exposure and control experiments. Based on our previous study result (^13,21^), a dark condition was chosen for the experiment. The exposure group *M. aeruginosa* was treated with 1.8 GHz and 40 V/m electromagnetic radiation in the dark for 24 h, temperature was 25 ± 1 °C, whereas the control group was not exposed to electromagnetic radiation and other conditions remained constant.

Electromagnetic radiation was generated using a vector signal generator (AgilentE8267DPSG) and a signal amplifier (AV38701E), and emitted from an antenna (ETS3180B). The antenna was placed at 24 cm above the sample area. The signal at the sample position was measured using an electromagnetic radiation analyzer (PMM8053B, Narta-STS, Italy) and a signal analyzer (AgilentN9030A). *M. aeruginosa* suspension were exposed to 1.8 GHz radio frequency electromagnetic radiation through a continuous sine wave, and at the position of the *M. aeruginosa* suspension, the radio frequency electromagnetic field strength was 40 V/m and the temperature was 25 °C. Experimental exposure device is the same as the device in the reference^21^.

Each experiment was repeated three times.

### Transcriptomic analysis

Three samples each from the treatment and control groups were sent to GENE DENOVO Company to extract RNA and Transcriptome sequencing. The ligation products were size selected by agarose gel electrophoresis, PCR amplified, and sequenced using Illumina HiSeqTM 4000 by Gene Denovo Biotechnology Co. (Guangzhou, China).

To get high quality clean reads, reads were further filtered by fastp (version 0.18.0). The edgeR package (version 3.12.1) (http://www.r-project.org/) was used to identify differentially expressed genes (DEGs) across samples or groups. We identified genes with a fold change ≥ 2 and a false discovery rate (FDR) < 0.05 in a comparison as significant DEGs. DEGs were then subjected to enrichment analysis of KEGG pathways (https://www.kegg.jp/kegg/kegg1.html). The calculated p-value has undergone FDR correction, taking FDR ≤ 0.05 as a threshold. Pathways meeting this condition were defined as significantly enriched pathways in DEGs.

### Determination of enzymes activity or content

Three samples each from the treatment and control groups were determinated total ATP synthase, H^+^K^+^-ATPase activity and ATP content.

Approximately 20 mL of *M. aeruginosa* suspension was taken, and algal cells were first collected by using a refrigerated centrifuge (Eppendorf 5810R). The cells were centrifuged at 10,000 r·min^-1^ and 4 °C for 3 min. Second, 1 mL of PBS or 0.9% saline solution and quartz sand were added to the suspension. Subsequently, a high-throughput tissue crusher (Shanghai Jingxin tiss-48) was used to crush the suspension for 6 min. Finally, the suspension was centrifuged at 10,000 r·min^-1^ for 5 min to obtain a test solution.

Total protein content, total ATP synthase activity, H^+^K^+^-ATPase activity, and ATP content were determined by kits (all kits used in the experiment were purchased from Nanjing Jiancheng Bioengineering Institute). Sample determination was performed in accordance with the kits’ instructions. The experiment was repeated three times.

Statistical analysis of the data was performed using SPSS 18 software. The significance analysis was performed using an independent sample t- test, and the data were expressed as mean ± SD). When* p* < 0.05, a significant difference was present between the control group and the exposed group.

## Supplementary information

Supplementary Information

## Data Availability

All data generated or analysed during this study are included in this published article.
