# Supplementary material for: Transcriptomic responses of Microcystis aeruginosa under electromagnetic radiation exposure
Source: Sci Rep. 2021 Jan 22;11:2123. doi: 10.1038/s41598-020-80830-z (PMC7822859; doi:10.1038/s41598-020-80830-z)
Supplement: Supplementary file 1 — Supplementary Information [file 41598_2020_80830_MOESM1_ESM.pdf]

Transcriptomic Responses of *Microcystis aeruginosa* Under Electromagnetic Radiation Exposure

Chao Tang, Ziyan Zhang, Shen Tian, Peng Cai *

Supplementary Table S1. Differentially expressed genes list

gene_name	log2(FC)	Pvalue	FDR
C789_RS04775	-2.784472325	0.002050028	0.031389198
C789_RS04780	-1.252449882	0.003347372	0.043795647
C789_RS04790	-1.367447603	0.000365923	0.0076554
C789_RS04815	-1.367447603	0.000365898	0.0076554
C789_RS04895	1.278370866	0.000276199	0.006241802
C789_RS04910	-1.620201733	9.32E-05	0.002662613
C789_RS05020	1.432959407	0.002542722	0.036058872
C789_RS05055	1.221000837	0.000229494	0.005388
C789_RS05400	-1.475244079	0.000908852	0.016323533
C789_RS05465	2.648227948	7.13E-08	6.28E-06
C789_RS05540	-1.500009717	8.00E-07	5.05E-05
C789_RS00060	1.013020997	0.003281189	0.043400757
C789_RS05655	-1.792393421	2.00E-05	0.000756827
C789_RS05820	-1.259213809	0.000434992	0.008880563
C789_RS06025	1.718611393	0.000135244	0.003572139
C789_RS06055	-1.086870526	0.000786162	0.014507945
C789_RS00065	-1.819048487	9.60E-09	1.27E-06
C789_RS06105	-1.271857699	0.001135734	0.019431621
C789_RS06240	-1.038625792	0.000585105	0.011342454
C789_RS06320	-1.369434951	0.000331984	0.007121637
C789_RS06395	1.057668773	0.000337682	0.007207298
C789_RS06455	-1.447799999	0.001554005	0.024875846
C789_RS06545	-1.326128363	3.02E-05	0.00102934
C789_RS06555	-1.496640775	1.50E-06	8.43E-05
C789_RS00070	-1.108805426	0.000106274	0.00297427
C789_RS06580	1.257860441	0.001655137	0.026394754
C789_RS00680	-1.334940502	0.000211583	0.005138802
C789_RS06835	-1.407681732	0.001779454	0.028071831
C789_RS07005	-2.317381485	1.18E-05	0.000477415
C789_RS07020	3.14503	0.000343654	0.007297897
C789_RS07030	4.196981858	9.33E-11	1.79E-08
C789_RS07210	1.618268094	0.000280904	0.006314357
C789_RS07355	1.137091595	0.003681036	0.047427004
C789_RS00760	-1.270057261	0.001013979	0.017854476
C789_RS00765	-1.213155599	0.000506482	0.010001835
C789_RS07530	1.392535501	0.003318969	0.043558889
C789_RS07535	-1.540536187	0.000203111	0.004990396
C789_RS07555	1.489313638	0.001061135	0.0183849
C789_RS07635	-2.632138827	1.85E-19	3.90E-16
C789_RS07640	-1.460472609	0.000311224	0.006744791
C789_RS07660	-2.024520276	4.18E-06	0.000193957
C789_RS07665	1.057050913	0.002363116	0.034145376
C789_RS07685	-2.706740123	3.30E-05	0.001107743
C789_RS08005	-1.404855178	2.73E-05	0.000937219
C789_RS08030	1.411988645	0.000111808	0.003088237
C789_RS08035	-1.20062641	0.001182285	0.020065605
C789_RS08135	12.00023479	0.003899209	0.049782656
C789_RS08185	1.33088951	7.23E-05	0.002197361
C789_RS08315	-1.912048697	2.16E-05	0.000802147
C789_RS08320	-1.780314395	1.99E-08	2.16E-06

C789_RS08325	-2.768325196	1.97E-13	1.04E-10
C789_RS08330	-2.996266315	1.72E-11	4.28E-09
C789_RS08360	-1.362778328	0.000224717	0.005335133
C789_RS08390	1.400664443	1.27E-05	0.000505467
C789_RS08475	1.53749099	0.000503982	0.009999182
C789_RS08505	-2.216807879	1.11E-06	6.66E-05
C789_RS00865	1.951309777	1.23E-06	7.11E-05
C789_RS08510	-2.584967825	8.49E-15	7.17E-12
C789_RS08515	-2.498473967	9.81E-12	2.59E-09
C789_RS08520	-2.180933406	1.39E-13	8.38E-11
C789_RS08525	-1.722437467	2.26E-07	1.65E-05
C789_RS08610	-1.180740521	0.000163744	0.004168574
C789_RS08615	-2.11271975	9.75E-12	2.59E-09
C789_RS08620	-2.54312043	1.49E-09	2.33E-07
C789_RS08650	1.811888294	0.000465945	0.009376593
C789_RS08710	-1.702392972	0.000762908	0.014258206
C789_RS00880	1.951309777	1.22E-06	7.11E-05
C789_RS08750	1.41798951	0.00015609	0.004046843
C789_RS00090	1.240443702	0.000724671	0.013732995
C789_RS00900	-1.723775241	0.003601692	0.046546639
C789_RS09160	1.410137916	0.00178023	0.028071831
C789_RS09180	1.888136186	2.98E-06	0.000148066
C789_RS09370	1.575218639	0.000541349	0.010542589
C789_RS00945	1.217333125	0.003398802	0.044331293
C789_RS09470	1.188184218	0.003291791	0.043400757
C789_RS00955	-1.517129427	0.000223961	0.005335133
C789_RS09795	1.965828094	3.98E-06	0.00018692
C789_RS09815	2.571313539	5.62E-09	7.66E-07
C789_RS09820	1.784958815	0.000268423	0.00609869
C789_RS10190	-1.612439799	2.31E-05	0.000833015
C789_RS10215	-1.459448203	0.00091964	0.016398307
C789_RS10235	1.640014332	0.000328204	0.007076484
C789_RS10275	-1.40359596	2.01E-05	0.000756827
C789_RS10280	-1.384087453	5.79E-06	0.000263134
C789_RS01040	1.470619032	0.003040757	0.041857457
C789_RS10325	1.352584799	0.002628217	0.037022821
C789_RS10460	-1.319187161	0.000199164	0.004922031
C789_RS10580	1.746447161	0.00023528	0.005493335
C789_RS10590	-1.396856113	0.002276017	0.033454268
C789_RS01070	1.188184218	0.003283859	0.043400757
C789_RS10850	1.188184218	0.003267122	0.043400757
C789_RS10885	1.011937587	0.001329359	0.021859428
C789_RS10895	-1.147808875	0.000248817	0.005744117
C789_RS10920	-1.692105457	2.62E-07	1.88E-05
C789_RS10980	-1.682016392	0.000600453	0.011586822
C789_RS11030	-1.31358363	8.21E-05	0.002375022
C789_RS01110	2.050024347	1.03E-08	1.32E-06
C789_RS11095	1.509814626	0.001061504	0.0183849
C789_RS01130	-1.299172195	1.86E-05	0.000727722
C789_RS11310	2.039191807	4.30E-11	9.55E-09
C789_RS01135	-1.226980482	0.000122906	0.003329487
pgi	-1.131473818	0.001305361	0.021718322
C789_RS11455	-1.529911881	2.44E-05	0.000859417
C789_RS11510	1.551936529	4.73E-07	3.22E-05
C789_RS11520	3.33960951	3.50E-07	2.42E-05
C789_RS11525	1.354454508	7.34E-05	0.002214567
C789_RS11530	-1.79505999	9.80E-05	0.002761435
C789_RS11540	-3.605372375	4.12E-16	5.81E-13

C789_RS11545	-2.124394765	1.50E-08	1.70E-06
C789_RS11550	-2.615627658	2.13E-15	2.25E-12
C789_RS11575	-1.392932265	2.25E-05	0.000821003
C789_RS11645	1.260075705	0.002048776	0.031389198
C789_RS11965	-1.094256102	0.003761494	0.048316339
C789_RS01205	-1.27670164	0.000215362	0.005200684
C789_RS12210	1.188184218	0.003289803	0.043400757
C789_RS12505	1.345736702	0.001840846	0.028812648
C789_RS12645	-1.022637198	0.000442159	0.008983473
C789_RS12650	-2.053829094	1.39E-08	1.67E-06
C789_RS01265	-1.810083523	0.00048336	0.009680954
C789_RS01270	-2.393765636	1.36E-07	1.09E-05
C789_RS01275	-1.770503268	7.32E-06	0.000318899
C789_RS01280	-2.550865989	7.98E-05	0.002325266
C789_RS01285	-2.260114852	9.70E-12	2.59E-09
C789_RS01290	-2.236097957	2.26E-12	8.70E-10
C789_RS01295	-1.553542648	2.04E-06	0.000106964
C789_RS12940	-1.353694946	1.12E-05	0.000460335
C789_RS13060	-2.281632342	5.58E-06	0.000256099
C789_RS13085	1.108692802	0.000162308	0.004157045
C789_RS13130	14.18275643	3.08E-06	0.000151119
C789_RS13135	1.311263822	3.55E-05	0.001172685
C789_RS13160	-1.12990766	2.43E-05	0.000859417
C789_RS13165	-1.451249373	2.08E-06	0.000106964
C789_RS13290	-1.509336051	0.001998433	0.030935451
C789_RS13370	1.521081918	0.000644895	0.012387854
C789_RS13465	-1.000573359	0.001061033	0.0183849
C789_RS01350	1.151052524	0.002087484	0.031651317
C789_RS13670	-2.751431612	2.02E-06	0.000106964
C789_RS13700	-1.28298826	1.19E-05	0.000477415
C789_RS13705	-1.089741726	4.95E-05	0.001573781
C789_RS13750	1.649630879	9.71E-08	8.37E-06
C789_RS13780	-1.471044057	2.18E-05	0.000802466
C789_RS01385	-1.405168849	7.68E-05	0.002284896
C789_RS13815	1.152345685	0.002089616	0.031651317
C789_RS13935	1.188184218	0.003284582	0.043400757
C789_RS13965	-12.85720347	2.14E-08	2.21E-06
C789_RS13975	-1.71470905	1.90E-06	0.000102911
C789_RS14145	1.382528264	0.001095051	0.018811735
C789_RS14365	1.032889256	0.000827469	0.015072781
C789_RS14405	1.306858047	0.001327716	0.021859428
C789_RS14415	1.100105867	0.000219165	0.005262461
C789_RS14460	-1.96017402	0.001765425	0.028047698
C789_RS14510	-1.216529589	0.00276617	0.038690408
C789_RS00145	-1.089532258	0.002317321	0.033768956
C789_RS14565	-2.294757894	0.000189254	0.004704642
C789_RS14610	-1.134232305	0.000161704	0.004157045
C789_RS14635	1.388698967	0.001864471	0.029074732
C789_RS14780	-1.795971333	3.04E-07	2.14E-05
C789_RS23150	-1.67183979	1.37E-05	0.000540656
C789_RS14905	-1.751675413	3.55E-08	3.41E-06
C789_RS14910	-1.284885455	3.24E-06	0.000157592
C789_RS15225	-1.31284403	7.45E-06	0.000321428
C789_RS15230	-1.198197962	8.87E-05	0.002550668
C789_RS15255	2.109774858	0.000378063	0.007870423
C789_RS15335	1.719591246	0.000401893	0.00832549
C789_RS15425	1.061693375	0.000116088	0.003185642
C789_RS15515	-1.132541063	7.81E-05	0.002292472

C789_RS15925	2.296238757	3.62E-05	0.001187277
C789_RS15950	1.156208829	0.002164383	0.032232359
C789_RS16055	2.116237643	1.02E-05	0.000422421
C789_RS16065	1.734867001	0.000128647	0.003462814
C789_RS16095	-1.206412075	0.000261823	0.005980887
C789_RS16100	-1.330938341	0.00082725	0.015072781
C789_RS16105	-1.801578864	4.24E-09	5.97E-07
C789_RS16110	-1.997766173	6.19E-12	2.18E-09
C789_RS16115	-1.749251078	3.49E-06	0.000167612
C789_RS16120	-2.433310728	6.01E-08	5.52E-06
C789_RS16125	-2.105211239	1.89E-05	0.000731485
C789_RS16130	-2.347634623	4.92E-14	3.46E-11
C789_RS16135	-2.161640786	1.35E-07	1.09E-05
C789_RS16140	-2.21102053	1.73E-12	7.31E-10
C789_RS16145	-1.655710853	0.00014643	0.003819837
C789_RS16150	-1.607674801	7.67E-06	0.000327616
C789_RS16165	-1.183179846	0.003115031	0.042669247
C789_RS16170	-1.124513407	3.38E-05	0.001126309
C789_RS16175	-1.876427953	2.09E-07	1.55E-05
C789_RS01620	1.300413507	0.000691333	0.013219783
C789_RS16340	-1.922608536	3.19E-05	0.001077659
C789_RS16465	1.335849995	0.000765881	0.014258206
C789_RS16570	1.224473174	0.002774064	0.038690408
C789_RS16740	1.747106211	5.09E-05	0.001604142
C789_RS16765	1.238309064	7.76E-05	0.002291968
C789_RS24765	2.398549376	0.002852434	0.039652579
C789_RS16940	1.251056012	0.000761205	0.014258206
C789_RS16945	1.263335476	4.01E-05	0.001304752
C789_RS16950	1.945791757	1.09E-06	6.66E-05
C789_RS17135	-1.162316304	5.61E-05	0.001743747
C789_RS17210	1.504170059	0.000229193	0.005388
C789_RS17260	1.57828303	0.000493085	0.009829129
C789_RS17470	-1.475272932	3.79E-06	0.000180193
C789_RS17475	-1.169907526	0.000304179	0.006690664
C789_RS17485	-1.101120584	0.00045378	0.009175469
C789_RS17620	1.915176303	5.14E-07	3.39E-05
C789_RS17760	1.591309085	1.10E-08	1.37E-06
C789_RS17785	-1.243261253	4.70E-05	0.001503284
C789_RS17930	1.768009246	0.002032381	0.031346142
C789_RS17935	1.188184218	0.003286177	0.043400757
C789_RS17940	-1.460073614	0.000187068	0.004677815
C789_RS18035	-1.050774354	0.002101021	0.031689377
C789_RS18170	-1.033838172	0.002419359	0.034658344
C789_RS18180	-1.634410084	1.53E-08	1.70E-06
C789_RS18185	-2.647268686	4.62E-13	2.17E-10
C789_RS18190	-2.223092737	8.96E-10	1.51E-07
C789_RS18380	1.792963017	0.000133956	0.003560375
C789_RS18650	1.90793715	2.60E-06	0.00013105
C789_RS18655	1.111730974	0.001300168	0.021717436
C789_RS18690	1.620248458	0.000868911	0.015759725
C789_RS18785	-1.253805619	2.69E-05	0.00093263
C789_RS18790	-1.808672611	1.67E-09	2.43E-07
C789_RS18795	-1.582831016	1.56E-07	1.20E-05
C789_RS18805	-1.090286151	0.00141343	0.023151769
C789_RS19015	-1.011325929	0.00030556	0.006690664
C789_RS19055	-1.14693789	0.000286458	0.006405139
C789_RS19155	1.90793715	2.59E-06	0.00013105
C789_RS19320	-1.724060402	1.12E-06	6.66E-05

C789_RS19435	1.978191267	8.67E-07	5.39E-05
C789_RS19485	1.156711559	0.002120445	0.031776596
C789_RS01975	-1.482769167	1.34E-06	7.64E-05
C789_RS01980	-1.966600124	5.97E-07	3.88E-05
C789_RS19855	2.653441259	1.59E-09	2.40E-07
C789_RS19860	2.612541226	8.44E-11	1.70E-08
C789_RS20000	1.068869183	0.000310813	0.006744791
C789_RS20035	-1.630511572	4.84E-07	3.25E-05
C789_RS20040	-1.422399332	0.000108692	0.003021916
C789_RS20050	-1.208125375	0.000722472	0.013732995
C789_RS20075	1.525458534	9.35E-06	0.000391167
C789_RS20080	1.659456649	1.47E-07	1.15E-05
C789_RS20085	1.969727929	5.48E-08	5.15E-06
C789_RS20215	2.1333508	2.92E-08	2.87E-06
C789_RS20510	-1.324975333	0.003119924	0.042669247
C789_RS20570	-1.179640722	0.000143818	0.003775005
C789_RS20725	-1.450783522	1.80E-06	9.91E-05
C789_RS20760	1.319363303	0.003160798	0.043088814
C789_RS20805	1.124759729	0.000250099	0.005744117
C789_RS20920	-1.129660908	0.002279893	0.033454268
C789_RS20970	1.240752381	0.00034848	0.007363373
C789_RS21005	1.765059385	0.00016617	0.004204995
C789_RS21010	1.761486393	0.001259769	0.021126126
C789_RS02150	-1.261649557	0.001549683	0.024875846
C789_RS02155	-1.778529593	8.10E-06	0.000342501
C789_RS02160	-2.488568261	8.42E-12	2.59E-09
C789_RS21470	-2.035955223	6.83E-11	1.44E-08
C789_RS21475	-1.21402288	0.001435665	0.023425176
C789_RS21485	-1.415781599	2.06E-06	0.000106964
C789_RS21590	2.922391734	2.13E-08	2.21E-06
C789_RS21710	-1.096231229	5.48E-05	0.001715773
C789_RS21760	1.60541399	0.001190813	0.020129501
C789_RS21780	-1.588555697	7.44E-07	4.76E-05
C789_RS21945	1.866200857	1.13E-07	9.58E-06
C789_RS21965	-1.613665863	6.81E-06	0.000303129
C789_RS21975	-1.562809009	6.37E-08	5.73E-06
C789_RS21985	-1.157522646	7.39E-05	0.002214567
C789_RS22135	-1.077254911	0.002289245	0.033475264
C789_RS22140	-1.280139799	0.001451118	0.023586255
C789_RS22195	2.672908819	0.00233686	0.033936674
C789_RS22240	-1.635375018	1.79E-06	9.91E-05
C789_RS22325	-1.08640659	0.000521196	0.010244534
C789_RS22365	1.216068108	0.000911584	0.016323533
C789_RS02285	-1.45674131	0.000174038	0.00437789
C789_RS02315	1.570352806	0.001224444	0.020615547
C789_RS00240	1.938618388	5.84E-05	0.001802921
C789_RS02425	-3.359096003	1.11E-09	1.81E-07
C789_RS02515	1.183029184	0.002375471	0.034145376
C789_RS02690	-2.067902302	2.60E-11	6.11E-09
C789_RS02695	-1.438896775	6.99E-06	0.000307537
C789_RS02715	-1.268802255	2.44E-05	0.000859417
C789_RS02825	-1.252528808	0.003518397	0.045609643
C789_RS02835	-1.209754703	0.001053388	0.0183849
C789_RS02955	1.650724381	0.002618628	0.037011108
C789_RS03030	-1.082304966	0.000929994	0.01651326
C789_RS03065	1.739921047	0.00329665	0.043400757
C789_RS03100	1.311626702	0.001092489	0.018811735
C789_RS03120	3.360883585	3.08E-10	5.67E-08

C789_RS03175	-1.284058346	0.000119117	0.003247676
C789_RS03345	-1.597335788	1.65E-07	1.25E-05
C789_RS03350	-1.150090483	6.08E-05	0.00186063
C789_RS03400	-1.906915122	8.28E-10	1.46E-07
C789_RS03460	-2.963608147	2.11E-05	0.000790591
C789_RS03480	1.430309336	9.42E-05	0.002672581
C789_RS03495	1.069751053	0.000407498	0.008400424
C789_RS00360	-1.373498198	0.00074471	0.014049758
C789_RS03570	1.299787732	4.27E-05	0.001376716
C789_RS00365	-2.849285554	4.11E-20	1.74E-16
C789_RS03635	-1.242483915	0.000806543	0.01481936
C789_RS03790	-1.521630891	0.00029966	0.006665077
C789_RS03795	-1.894951208	1.43E-08	1.68E-06
C789_RS03970	1.584777326	0.000240326	0.005580322
C789_RS03985	1.391773337	0.002107126	0.031689377
C789_RS04005	-1.423575944	2.61E-05	0.000910523
C789_RS04010	-1.905508251	1.90E-05	0.000731485
C789_RS04015	-1.990925534	1.16E-07	9.62E-06
C789_RS04020	-1.754884296	6.25E-06	0.000281159
C789_RS04095	-1.08290009	0.000525235	0.010276117
C789_RS04150	-1.1954486	0.000304334	0.006690664
C789_RS04205	-1.437418322	0.000130462	0.003489438
C789_RS04240	-1.800530014	2.27E-08	2.29E-06
C789_RS04440	1.220214708	0.000206761	0.005050705

Table S1 Note:

Log2 (FC) represents the logarithm of genes with a fold change between E and C, with 2 as the bas

If | log2FC |> 1 and false discovery rate (FDR) <0.05, the difference is significant.
